# How gender-sensitive is nursing care in hospitals? Results of a national questionnaire survey in cardiology in Germany

**DOI:** 10.1016/j.ijnsa.2026.100631

**Published:** 2026-07-15

**Authors:** Judith Mollenhauer, Sophia Sgraja, Martina Kloepfer, Ute Seeland, Volker E. Amelung, Clarissa Kurscheid

**Affiliations:** a Institute for Epidemiology, Social Medicine and Health Systems Research, Hannover Medical School (MHH), Carl-Neuberg-Str. 1, 30625, Hanover, Germany; b figus – Research Institute for Health- and System Design, Domstr. 55-73, 50668, Cologne, Germany; c Institute for Gender Health, Wartburgstr. 11, 10823, Berlin, Germany; d Medical Faculty/ University Hospital Magdeburg Otto von Guericke, Leipziger Str. 44, 39120, Magdeburg, Germany; e German Society of Gender-specific Medicine (DGesGM e.V.), Potsdam, Germany

**Keywords:** Gender-sensitive nursing care, Gender sensitivity, Nurses, Quantitative survey, Questionnaire, Hospitals, Germany

## Abstract

**Background:**

Gender-sensitive personalized nursing care is part of patient-centered care and is a prerequisite for high-quality care. Although nursing expert standards include evidence-based gender-sensitive personalized nursing care recommendations, the degree of implementation in hospitals is unknown.

**Objectives:**

This study investigates the extent of gender sensitivity among nursing professionals, their level of knowledge regarding gender-sensitive personalized nursing care, and the perceived degree of institutional implementation of gender-sensitive personalized nursing care in hospital settings by nurses.

**Design:**

Quantitative questionnaire survey

**Settings:**

German hospitals with cardiology wards or internal medicine wards (with a cardiology department).

**Participants:**

Nurses - each eligible German hospital was invited to participate in the study by e-mail.

**Methods:**

The quantitative nationwide questionnaire survey was conducted online from May 2024 to February 2025. A total of 111 questionnaires were included in the evaluation. Gender sensitivity was assessed using the validated Nijmegen Gender Awareness in Medicine Scale (N-GAMS). In a previous screening of the expert standards, gender-sensitive content and measures were identified and formulated into self-developed questions.

**Results:**

Nurses are moderately gender-sensitive (M = 3.46, SD=0.75) and perceive gender-sensitive personalized nursing care implementation in hospitals as 70% implemented (M = 0.70, SD=0.15). In general, nurses with higher gender sensitivity reported lower levels of implementation in hospital wards. University hospitals (p < 0.001) and hospitals with >800 beds (p < 0.05) show a lower degree of gender-sensitive personalized nursing care implementation than hospitals with ≤800 beds. Nurses who are born in Germany (p < 0.01), in comparison to nurses born in another country, show a higher gender sensitivity.

**Conclusion:**

The results show moderate gender sensitivity among nursing professionals and implementation in practice with potential for improvement. Training for nursing professionals is needed to raise awareness and close knowledge gaps related to gender-sensitive personalized nursing care. At an organizational level, change agents, gender balance, internal training, or quality circles are needed to ensure that hospitals are gender-sensitive, for example, in patient care, room occupancy, menu selections, and shift planning. Expert standards should include more practice-oriented guidance on translating theory into daily practice. Nursing professionals could serve as multipliers for patients, disseminating knowledge about gender-specific symptoms and increasing health literacy.

**Contribution of the paper**

What is already known about the topic?

• Gender-sensitive personalized nursing care is implemented in nursing expert standards and is part of patient-centered care
• Patient-centered care is a prerequisite for high-quality care

What this paper adds

• Evaluation of the implementation of gender-sensitive personalized nursing care in German hospitals (status quo)
• An assessment of the gender sensitivity among nurses

Alt-text: Unlabelled box dummy alt text

## 1 Background

The consideration of gender in patient care is part of gender-sensitive personalized nursing care. Gender-sensitive nursing care considers “sex” - the specific biological and physiological characteristics of females, males, or intersex, and “gender” - the socially constructed characteristics of women and men in patient nursing care (Regitz-Zagrosek, 2012; World Health Organization, 2025). Further “factors of diversity” are age, sociocultural and socioeconomic status, mental health, physical performance, religion, ethnicity, culture, and sexual identity (Deutsche Gesellschaft für Geschlechtsspezifische Medizin (DGesGM) e.V., 2025; Mollenhauer et al., 2026). Nurses must be sensitized to and aware of these additional, relevant aspects of patient individuality in order to enhance patient well-being and the quality of care in clinical practice (Besharati and Kalaleh, 2024; Jandaghian-Bidgoli et al., 2025; Lopes-Júnior, 2021; Yang et al., 2023). Van Servellen defined in the 1980s the traditional concept of individualized care in nursing practice as nurses’ acknowledgment of the patient's unique characteristics and clinical condition, acknowledging the patient as an embedded member of their familial and social context, and exhibiting a professional commitment to actively listening and responding to patient-expressed concerns (van Servellen, 1988).

Professional training (e.g., academic studies) improves the quality of patient care (German Science And Humanities Council, 2023). In Germany, nurses complete a three-year vocational school training program to become certified nurses. In addition, individuals may complete a one-year training program to become a nursing assistant or pursue a degree in nursing science at the bachelor's or master's level. There are 1.8 million nursing staff working in Germany. Around 62% are certified nurses, while 38% are nursing assistants. As studies in Germany have only been state-funded since 2024 due to changes in the law, the number of bachelor's degree graduates is very low (2024: 8.600 nurses with an academic degree (Rottenkolber and Thiele, 2025)). Germany does not meet the recommendation of the German Science and Humanities Council, which states that 10 to 20% of nurses should have a university degree (German Science And Humanities Council, 2023). Compared to Germany, almost all nursing staff in the UK are academically qualified, while in the US, the figure is around 72% (minimum bachelor's degree) (American Association of Colleges of Nursing, 2024). According to the framework plans for nursing training in Germany (§ 53 Nursing Professions Act (PflBG)), special requirements of the “age/life/development phases and the living environment as well as formative biographical, cultural, and religious aspects of the people being cared for must be taken into account, while preserving the self-determination of the people being cared for.” (Bundesinstitut für Berufsbildung, 2024). Furthermore, individual-related aspects are stated, such as the consideration of gender, sexual diversity, or ethics; sex-specific aspects do not occur. Nevertheless, gender- and age-specific characteristics must be considered in patient care according to § 2b of the Social Security Code V. Furthermore, further education for nurses is not mandatory or regulated at the federal level. Nevertheless, nurses must work in accordance with the latest scientific findings (§ 113 Social Security Code XI). Training sessions on topics such as LGBTQ+ issues, legal matters, or communication skills can be offered in-house or by the professional association (Deutscher Berufsverband für Pflegeberufe, 2026). In addition, there are training programs to qualify nurses for new tasks (e.g., intensive care, palliative care, wound management). These are voluntary and can help advance their career.

The gender awareness of healthcare providers can be evaluated using the “Nijmegen Gender Awareness in Medicine Scale” (N-GAMS). The scale is divided into three subscales: Gender sensitivity, gender role ideology towards patients, and gender role ideology towards doctors. Verdonk et al. developed the N-GAMS in 2008 to quantify the gender awareness of medical students (Verdonk et al., 2008). Following surveys used the N-GAMS across the world in translated versions, researchers validated the scale and applied it to different healthcare professionals, nurses, and students (Aliri et al., 2022; Gattino et al., 2024; Goussault-Capmas et al., 2024; Labaka et al., 2023; Prego-Jimenez et al., 2022; Shamasneh et al., 2023). However, the degree of gender sensitivity of nursing professionals in Germany is not known yet.

Evidence-based gender-sensitive personalized nursing care is part of high-quality and patient-centered nursing care and is considered in German nursing expert standards. Expert standards are evidence-based quality standards recognized in German-speaking countries (Germany, Austria, Switzerland) that define what constitutes good nursing care. They are organized into three categories: structural quality, process quality, and outcome quality. There are 12 expert standards on the following topics: decubitus prophylaxis, patient discharge management, pain management, fall prevention, continence promotion, care of chronic wounds, nutrition management, patients with dementia, oral health, skin integrity, physiological birth, and promotion of mobility which were developed by expert working groups within the German Network for Quality Development in Nursing Care (Deutsches Netzwerk für Qualitätsentwicklung in der Pflege) (Deutsches Netzwerk für Qualitätsentwicklung in der Pflege, 2025a). Expert standards are indication-specific and, unlike medical guidelines, are not structured according to medical specialties. The content on gender-sensitive personalized nursing care is included in each expert standard and is presented as expert knowledge, prevalence/incidence figures, or specific implementation measures. Recommendations regarding the implementation of gender-sensitive personalized nursing care are provided in the expert standards on patient discharge management, continence promotion, and nutrition management.

In Germany, there are a total of 1875 hospitals with 476,924 beds (2023) (Kassenärztliche Bundesvereinigung, 2023). Of these, 48% are publicly funded, 32% are non-profit, and 20% are privately funded. 16.4% of all hospitals have ≥ 500 beds, including university hospitals. 32.2% of hospitals have 200–499 beds, and 51.4% of hospitals have <200 beds. Approximately 30% of all beds are in internal medicine (including a department of cardiology) (Destatis, 2022).

Cardiology was the first medical specialty to identify sex-based differences in anatomy, physiology, diagnostic approaches, and treatment (Legato and Colman, 1991). As part of the health research project HeartGap (Gender health gaps in guideline-based inpatient cardiovascular medical and nursing care and implementation strategies to reduce the gap), funded by the Innovation Fund of the Federal Joint Committee (funding number: 01VSF22030), the implementation status of gender-sensitive personalized care and influencing factors between 2023 and 2025 is investigated (Sgraja et al., 2024). This final part of the study quantitatively examines nursing professionals’ gender sensitivity and the perceived degree of gender-sensitive personalized nursing care implementation in hospital cardiology using a questionnaire survey.

## 2 Methods

### 2.1 Inclusion and recruitment

In preparation for the questionnaire survey, all eligible hospitals (n = 994) in Germany were successively contacted via e-mail and invited to participate. The inclusion criteria for recruiting hospitals were: 1) hospitals in Germany, listed in the German hospital register, and 2) hospitals with a cardiology specialty or specialty of internal medicine with a department of cardiology.

The invitation e-mail consisted of a cover letter and access to the online survey (link and QR code). As part of a reminder strategy, the e-mail containing the links to the online survey was sent again successively. The quantitative nationwide questionnaire survey was conducted online from May 2024 to February 2025 using an online questionnaire on the LimeSurvey platform (https://www.limesurvey.org/de). The inclusion criteria were: nurses and nurses in training from the cardiology ward or internal medicine with a department of cardiology; exclusion criteria were: nurses from another ward or specialty.

Participation was anonymous and voluntary. Participants agree to a consent form before attending. The study is registered in the German Register of Clinical Studies under study number DRKS00031317 (registration date: 24.02.2023). The study is considered "uncritical" and has been approved by the local ethics committee of the Hannover Medical School in Hanover, Germany (approval number 1075_BO_K_2023).

### 2.2 Questionnaire

The online questionnaire is divided into three parts. The first part includes questions about socio-demographics such as sex, age, migration background, graduation, professional qualification, and years of professional experience as a nurse. Besides, questions about the hospital´s level of care (hospital with ≤500 beds, hospitals with 501–800 beds, hospital >800 beds, teaching / research hospital with >800 beds) and region of hospital (rural community and city with <100,000, and large city with ≥100,000 inhabitants) were asked. The second part consists of the N-GAMS subscale gender sensitivity with a five-point Likert-type scale ranging from “strongly disagree” = 1 to “strongly agree” = 5. The N-GAMS scale was available in German language (unpublished thesis by Veronika L. Landerer 2010) and was provided to us personally by Verdonk (N-GAMS-developer). In the N-GAMS scale, the term “physicians” was replaced by the term “nurses” for this study (Aliri et al., 2022; Prego-Jimenez et al., 2022). Due to the lack of comprehensibility of question 1, as shown in previous N-GAMS studies, it was removed from the scale sensitivity (13 items) (Steinböck et al., 2015; Wortmann et al., 2023). As a result, the instrument represents a modified version of the original scale, which may limit direct comparability with studies using the full version. The reliability of the modified scale is acceptable (Crohnbach´s alpha= 0.768); Factor analyses were not conducted. Items two to eleven and 13 are reversed items. The third part of the questionnaire consists of questions on gender-sensitive personalized nursing care implementation and knowledge (14 items). Before conceptualizing the questionnaire, the expert standards (decubitus prophylaxis, patient discharge management, pain management, fall prevention, continence promotion, care of chronic wounds, nutrition management, patients with dementia, oral health, skin integrity, and promotion of mobility) were screened on gender-sensitive personalized nursing care contents. The selected search terms were: sex, gender, woman, women, man, men, female, and male. Furthermore, the expert standards were screened for text passages considering diversity factors like age, ethnicity, religion, culture, and transgender. Relevant content was identified in ten of the eleven standards examined, particularly in the standard on urinary continence. In the appendix “Summary of expert standards regarding gender-sensitive contents”, there is a table with an overview of all identified gender-sensitive personalized nursing care contents of the expert standards. Three of the expert standards (patient discharge management, continence promotion, and nutrition management) contain explicit measures for implementation in healthcare institutions. These contents were summarized, formulated as questionnaire questions with predefined answer options, discussed in the research team, and finalized. For reliability analysis, Cronbach’s alpha was calculated to assess the internal consistency of the subscale for implementation, which consists of twelve questions. The internal consistency of the questionnaire is acceptable, with Cronbach’s alpha = 0.767. Finally, the open-ended optional questions were asked:

• “Women are more likely to present with symptoms such as abnormal fatigue, shortness of breath, and nausea than men with acute myocardial infarction.”,
• “In your opinion, to which extent is gender-sensitive care (e.g., respecting patients’ privacy, respecting for religious affiliation, e.g., space to pray or consideration of the migration background, e.g., language barriers or nutritional habits) implemented on this ward?” and
• “In your opinion, what role does the patient's gender play in nursing-patient communication (optional)?”.

Three volunteer nurses in further training participated in a pretest using the read-aloud and think-aloud methods. The questionnaire was reviewed for clarity, completeness, and practicality. The feedback was incorporated into the questionnaire, and the questionnaire was finalized.

### 2.3 Data preparation

The data were downloaded from the online questionnaire platform, LimeSurvey, and checked by a researcher. Only questionnaires containing at least a completed personal section and the N-GAMS section were considered for data analysis. Twenty-three incomplete questionnaires were excluded. One more was excluded because it was a straightliner (Runkler, 2025). In this straight-lining questionnaire, only the first answer option was ticked. Following the exclusion of 24 questionnaires, 111 were included in the analysis.

In preparation for the study, an a priori calculation regarding strata distribution based on region and hospital level was conducted (Sgraja et al., 2024). The data set was verified concerning the strata distribution, and absolute frequencies were counted. Due to the smaller sample size in comparison to a priori calculation (Sgraja et al., 2024) and the deviating distribution, the aim was to weight the data to avoid distortions in the results. For this, the a priori calculation and absolute frequencies of the survey were divided by the number of the entire sample. To calculate the weighting per strata, the relative frequency of the a priori calculation was divided by the relative frequency of the survey data. The weights per stratum are shown in Table 1. The survey data set was weighted for descriptive statistics. As the model accounted for all covariates related to unequal selection probabilities and selective nonresponse, consistent with the approach used in the present study, additional weighting should not be performed for inferential statistics. (Kiesl, 2022).

**Table 1:** Weights per strata based on region and hospital level regarding bed size.

Strata no.	A priori calculation (%)		Survey (%)	Weights
**1**	11	18		0.61
**2**	3	8		0.38
**3**	3	17		0.18
**4**	1	2		0.50
**5**	9	13		0.69
**6**	5	6		0.83
**7**	19	17		1.12
**8**	48	17		2.82
**9**	1	2		0.50

Note: Strata no 1 = Teaching/research hospital in large city, strata no 2 = Teaching/research hospital in city, strata no 3 = Hospital with > 800 beds in large city, strata no 4 = Hospital with > 800 beds in city, strata no 5 = Hospital with 501–800 beds in large city, strata no 6 = Hospital with 501–800 beds in city, strata no 7 = Hospital with ≤ 500 beds in large city, strata no 8 = Hospital with ≤ 500 beds in city, strata no 9 = Hospital with ≤ 500 beds in rural community.16.

### 2.4 Statistical methods and content-based qualitative evaluation

The entire analysis was performed using the statistics software IBM SPSS version 29. First, the absolute and relative frequencies were calculated for each question. Second, the gender sensitivity (N-GAMS) and degree of implementation were determined using a mean value index. Some items had to be recoded for this purpose. Spearman correlation was used to examine the relationship between the degree of implementation of gender-sensitive personalized nursing care and gender sensitivity. Multiple linear regression was used to examine the relationship between the variables of sex, age, migration background, level of care, region, and the degree of implementation, as well as gender sensitivity. In preparation for the multivariate analyses and due to the exploratory character of the research, a backward elimination was conducted in the preliminary analysis with all independent variables of the nurses and the nurses’ workplace. These were: age, country of birth, sex, graduation, professional qualifications, professional experience in years, size of hospital, and region of hospital. Variables with a p-value above 0.1 were excluded from the regression model. Given the exploratory nature of the context and the lack of a theoretical foundation, backward elimination was used to achieve an optimal model. All available predictors were included in the backward elimination process, and variables with low influence were eliminated (p > 0.1). This method has the advantage of improving model interpretability and reducing multicollinearity. Multicollinearity was checked and verified in the full model, including all relevant predictors. Since all predictors have a VIF value close to 1, multicollinearity can be ruled out. Furthermore, homoscedasticity and linearity were confirmed, as well as normal distribution was assessed in the full model by examining the residuals in plots (scatter plot of the residuals and histogram).

A content-based qualitative evaluation was conducted with open questions at the end of the questionnaire.

## 3 Results

### 3.1 Sample description

In total, 135 nurses completed the online questionnaire, and 111 questionnaires were included in the evaluation. Incomplete questionnaires were excluded (at least the N-GAMS had to be completed in full). Table 2 shows the characteristics of the 111 nurses (part A in the questionnaire). More nurses were female (n = 77, 70.0%) than male (n = 33, 30.0%). The mean age of nurses was 39.6 (SD= 11.9), and the range was between 18 and 70. Most participants (79.2%) were born in Germany. All of the nursing professionals had a school certificate, and the majority had a high school diploma (50.0%) or a certificate of secondary education (44.2%). Most of the participants had professional qualifications (93.9%). 17.7% had a university degree, 76.2% had completed vocational training, and 2.8% of nurses were in vocational training. The mean of years that professionals worked as nurses was 18.9 (SD= 12.43), and the range is between 0 and 53 years. About two-thirds of the nurses worked in hospitals with ≤ 500 beds (68.3%). Most of the participants worked in hospitals in cities (57.6%), followed by hospitals in large cities (41.5%), and hospitals in rural communities (0.9%).

**Table 2:** Characteristics of the participating nurses.

	M	SD	n (%) (total n = 111)	Range
**Gender**			110 (100)	
Female			77 (70.0)	
Male			33 (30.0)	
**Age (years)**	39.6	12.0	108 (100)	18–70
18–30			28 (25.9)	
31–40			30 (27.9)	
41–50			28 (25.6)	
51–60			19 (17.6)	
61–70			3 (3.0)	
**Country of birth**			110 (100)	
Germany			87 (79.2)	
Other			23 (20.8)	
**Graduation**			109 (100)	
High school diploma			54 (50.0)	
Certificate of secondary education			48 (44.2)	
Lower secondary school leaving certificate			6 (5.2)	
Other			1 (0.6)	
**Professional qualification**			106 (100)	
University degree			19 (17.7)	
Completed vocational training			80 (76.2)	
In vocational training			3 (2.8)	
Other			4 (3.3)	
**Years of profession**	18.9	12.4	109 (100)	0–53
<10			39 (35.6)	
10–20			22 (20.5)	
21–30			24 (21.9)	
>30			24 (22.0)	
**Level of care**			110 (100)	
Teaching/ research hospital with > 800 beds			16 (14.3)	
Hospital with > 800 beds			4 (4.0)	
Hospital 501–800 beds			15 (13.4)	
Hospital with ≤ 500 beds			75 (68.3)	
**Population size**			109 (100)	
Large city			45 (41.5)	
City			63 (57.6)	
Rural community			1 (0.9)	

M = mean; SD = standard deviation; weighted data.

Table 3 shows the means and standard deviations of each item of the N-GAMS scale for nurses (part B in the questionnaire). The mean value index is 3.46 (SD= 0.75, min.= 1.46, max.= 4.77), and it ranges between 1 and 5, where 1 = low gender sensitivity and 5 = high gender sensitivity. The mean of 3.46 shows a moderate gender sensitivity in the sample. The mean of gender sensitivity is highest in the statements on the biological differences between women and men (Item 2: M = 3.94; SD= 1.26), gender sensitivity as an important issue (Item 8: M = 3.97; SD= 1.21) and, too small differences between women and men to take into account (Item 11: M = 3.90; SD= 1.18). The mean of gender sensitivity is lowest in the statements on gender in case of non-sex-specific health disorders (Item 3: M = 2.80, SD= 1.26), same treatment of women and men (Item 7: M = 2.65, SD= 1.54), and communication between nurses and patients and impact of patient´s gender (Item 9: M = 2.74, SD= 1.37).

**Table 3:** Results of the N-GAMS scale reformulated for nurses (scale range 0–5).

No.	Item	M	SD
1	Nurses’ knowledge of gender differences in illness and health increases quality of care.	3.60	1.38
2	Nurses should only address biological differences between men and women.	3.94	1.26
3	In non-sex-specific health disorders, the sex/ gender of the patient is irrelevant.	2.80	1.26
4	A nurse should confine as much as possible to biomedical aspects of health complaints of men and women.	3.59	1.23
5	Nurses do not need to know what happens in the lives of men and women to be able to deliver medical care.	3.71	1.29
6	Differences between male and female nurses are too small to be relevant.	3.83	1.15
7	Especially because men and women are different, nurses should treat everybody the same.	2.65	1.54
8	Nurses who address gender differences are not dealing with the important issues.	3.97	1.21
9	In communicating with patients, it does not matter to a nurse whether the patients are men or women.	2.74	1.37
10	In communicating with patients, it does not matter the nurse is a man or a woman.	2.83	1.36
11	Differences between male and female patients are so small that nurses can hardly take them into account.	3.90	1.18
12	For effective treatment, nurses should address gender differences in etiology and consequences of disease.	3.74	1.05
13	It is not necessary to consider gender differences in presentation of complaints.	3.61	1.39

M = mean; SD = standard deviation; weighted data.

### 3.2 Univariate analyses

Table 4 shows the means and standard deviations of the items of the third part of the questionnaire. The dimension “Gender sensitivity in hospital” subsumes items based on the expert standards of patient discharge management, continence promotion, and nutrition management, focusing on gender sensitive content. Each item's score was recoded between 0 and 1 for building the mean value index. The mean value index of the 12 items is 0.70 (SD= 0.15; min= 0.17 to max= 1) and shows a well-rated gender sensitivity in hospitals perceived by the nurses. The mean index is an initial estimate designed to illustrate the extent to which gender-sensitive personalized nursing care is embedded in hospital structures. Given the exploratory nature of the study, the value should not be interpreted as theory-based or exhaustive.

**Table 4:** Scores of items of the dimensions gender sensitivity in hospital regarding expert standards – patient discharge/continence promotion/nutrition management, knowledge of gender differences, and perceived degree of implementation on the ward.

Item	M	SD	Range	n
**Expert standard patient discharge**				
Recording age on admission to hospital	0.99	0.09	0–1	100
Recording ethnicity on admission to hospital	0.47	0.42	0–1	100
Recording sex on admission to hospital	0.98	0.13	0–1	100
Recording chronic diseases on admission to hospital	0.99	0.07	0–1	100
**Expert standard continence promotion**				
Same-sex nursing care	3.08	0,80	1–4	92
Illustrative materials for consulting of incontinence	2.43	1.04	1–4	80
Gender-sensitive incontinence aids	3.26	0.80	1–4	96
Garbage can on men´s toilets	2.95	1.11	1–4	89
Female and male nurses on shift	2.12	1.14	1–4	90
Toilet aids for the female anatomy	2.40	1.15	1–4	92
Toilet aids for the male anatomy	2.92	1.04	1–4	92
**Expert standard nutrition management**				
Person-centered meal selection	3.08	0.86	1–4	96
**Knowledge about gender differences**				
Pain	0.90	0.30	0–1	99
Fall prevention	2.57	0.99	1–4	70
Symptoms of myocardial infarction	0.86	0.35	0–1	100
**Perceived degree of implementation**				
Implementation on the ward	2.91	0.81	1–4	91

Note. The data is weighted according to hospital level and region.

In addition, the nursing professionals were asked three questions about their gender-sensitive knowledge of gender differences. Ninety percent of nurses know that estrogens and the genetics of women are influencing factors of pain perception and prevalence (M = 0.90, SD= 0.30). Nurses sometimes initiate measures for fall prevention if prostate cancer patients get androgen receptor inhibitors (M = 2.57, SD= 0.99). Eighty-six percent of nurses know that myocardial infarction can present more often with atypical symptoms in women than in men (M = 0.86, SD= 0.35).

### 3.3 Multivariate analyses of factors related to the perceived degree of gender-sensitive personalized nursing care implementation

Table 5 presents the results of the multivariate linear regression analysis of gender-sensitive personalized nursing care implementation and its subfactors on age, migration background, and hospital size. The dependent variable is the mean value index of the questions relating to the expert standard and the contents of the gender-sensitive personalized nursing care. University hospitals (b= −0.139, p < 0.001) and hospitals with >800 beds (b= −0.087, p < 0.05) had a worse degree of gender-sensitive personalized nursing care implementation than hospitals with ≤800 beds. The age of the nurses (b= −0.002, p > 0.05) and the country of birth (b= −0.076, p > 0.05) are not related to the dependent variable.

**Table 5:** Results of the multiple linear regression analysis of the influencing factors of gender-sensitive personalized nursing care implementation.

Predictor	B	β	CI (95%)		p
(Intercept)	0.866		[0.762;	0.970]	<0.001
Country of birth (1=Germany)	−0.076	−0.183	[−0.152;	0.000]	0.051
Age	−0.002	−0.155	[−0.004;	0.000]	0.091
University hospitals^a^	−0.139***	−0.393	[−0.206;	−0.072]	<0.001
Hospitals with > 800 beds^a^	−0.087*	−0.214	[−0.162;	−0.011]	0.024
R^2^ / Adj. R^2^	0.253 / 0.221				
n	100				

*Note.* B= unstandardized regression coefficient; β= standardized coefficient. The data is unweighted.

a References: Hospitals with 501–800 beds and hospitals with ≤500 beds. * p < 0.05, ** p < 0.01, *** p < 0.001.

Table 6 shows the results of the multiple linear regression analysis of the N-GAMS score and its subfactors on country of birth, graduation (A-levels), and professional experience in years. The dependent variable is the mean value index of the N-GAMS scale. Nurses who are born in Germany (b = 0.638, p < 0.01), in comparison to nurses born in another country, show a higher gender sensitivity. The A-level graduation (b = 0.272, p > 0.05) and the professional experience in years (b = 0.010, p > 0.05) are not related to the dependent variable.

**Table 6:** Results of the multiple linear regression analysis of the influencing factors of N-GAMS.

Predictor	B	β	CI (95%)		p
(Intercept)	2.556		[2.157;	2.976]	<0.001
Country of birth (1=Germany)	0.638**	0.309	[0.262;	1.014]	0.001
A-levels^a^	0.272	0.172	[−0.018;	0.561]	0.065
Professional experience in years	0.010	0.166	[−0.001;	0.022]	0.080
R^2^ / Adj. R^2^	0.154 / 0.129				
n	107				

*Note.* B= unstandardized regression coefficient; β= standardized coefficient. The data is unweighted.

a References: Certificate of secondary education, Lower secondary school leaving certificate, no school-leaving certificate, other *p < 0.05, **p < 0.01, ***p < 0.001.

### 3.4 Correlation between gender sensitivity (N-GAMS score) and perceived degree of implementation

The Spearman correlation result shows a significant medium inverse correlation between nurses’ gender sensitivity (N-GAMS variable) and the degree of perceived implementation at the ward (imp1 variable) (r= −0.360, p < 0.05, n = 109). This means that, in general, nurses with higher gender sensitivity have noticed less implementation in hospital wards (Table 7).

**Table 7:** Correlations.

	Gender sensitivity x Implementation of gender-sensitive personalized nursing care	Implementation score x Implementation of gender-sensitive personalized nursing care
B	−0.360***	0.415***
p	<0.001	<0.001
n	109	109

*Note.* B= unstandardized regression coefficient; The data is weighted according to hospital level and region.

*p < 0,05. **p < 0,01. ***p < 0001.

Another Spearman correlation result shows a significant moderate correlation between the degree of perceived implementation at the ward and general implementation in the hospital (mean value index of the questions relating to the expert standard and the contents of the gender-sensitive personalized nursing care) (r = 0.415, p < 0.001, n = 109). This implies that, in general, a well-executed implementation of gender-sensitive personalized nursing care tends to be positively perceived by nurses on the ward (Table 7).

### 3.5 Open question about nurse-patient communication

Thirty-four of the participating nurses responded to the last optional open question regarding gender and nurse-patient communication. A content-based qualitative evaluation was conducted. Approximately 50% of the participants did not recognize that gender influences communication between nurses and patients. In the following are a few quotes from the nurses:

*“In my opinion, it doesn't matter; a person is a person.” (Questionnaire 72).*

*“The patient is a number and is “processed”.” (Questionnaire 35).*

*“Everyone should be treated with equal respect. For me personally, gender makes no difference unless the patient expresses a preference for being treated by a male or female practitioner (whether for religious or other reasons). Our goal is the well-being of our patients.” (Questionnaire 46).*

Nurses wrote that same-sex communication between female nurses and female patients, or male nurses and male patients, can be facilitative by building trust and openness, or desired by patients with specific religious backgrounds.

*“Partially plays a major role - changes the basis of trust.” (Questionnaire 44).*

One nurse responded that same-sex communication is helpful at initial patient contact; subsequently, further factors other than gender are relevant for nurse-patient communication.

*“The gender of nurses and patients can play a role in communication, especially when it comes to topics that are taboo, disgusting, or shameful. However, this seems to play a greater role primarily during initial contact and while building a relationship. Beyond that, in my experience, the personal preferences of patients and building a good relationship are much more important than reducing everything to gender.” (Questionnaire 9).*

## 4 Discussion

Evidence-based nursing care is based on expert standards in Germany. A preliminary screening for this study showed that gender-sensitive content is included in the expert standards. Gender-sensitive care is a component of personalized care and a further step toward enhancing the quality of care (Besharati and Kalaleh, 2024; Jandaghian-Bidgoli et al., 2025; Lopes-Júnior, 2021; Yang et al., 2023). The content of gender-sensitive personalized nursing care is presented in expert standards as expert knowledge, prevalence and incidence figures, or specific implementation measures. Nurses’ knowledge about gender-sensitive personalized nursing care, their gender sensitivity, and perceived implementation of gender-sensitive personalized nursing care in cardiology were evaluated in this explorative survey.

Although gender-sensitive personalized nursing care has not been considered in a structured approach in professional training, development of expert standards, or in everyday hospital practice (Mollenhauer et al., 2025), this study shows moderate results of nurses’ gender sensitivity and gender-sensitive personalized nursing care implementation.

The mean value index (M_N-GAMS_= 3.22) and single means are comparable to the results of the Spanish study from Aliri et al. (M_S-NGAMS_= 3.25), where nursing students were asked the N-GAMS scale tailored towards nurses (Aliri et al., 2022). It should be emphasized that 14 questions were used for the gender sensitivity scale of the N-GAMS in comparison to this study, which used 13 questions. Both surveys showed low mean values for the item addressing communication between nurses and patients and the influence of gender. The open-ended question at the end of the questionnaire in the present study aimed to explore the reasons for and against same-sex communication between patients and nurses. A quarter of the sample responded to the optional question, and one-half of them did not see any relevance. The other half pointed out that same-sex communication strengthens the trust and openness of patients and could be desired by patients for personal reasons. In addition, a few nurses responded that they try to implement same-sex nursing care and communication if required. Due to staffing shortages in hospitals and the significantly higher number of female nurses compared to male nurses, providing same-sex nursing care is not always feasible in practice. Furthermore, the patients’ perspectives and knowledge should be evaluated and considered to patients’ needs during hospitalization. This aspect constitutes the next phase of the German HeartGap study, for which a publication is currently in preparation. Teunissen et al. investigated in 2015 which gender differences in quality of patient-centered care experience during hospital stay in the Netherlands exist. They identified that women assess hospital care significantly lower and experience more imperfections in nursing care than men. Additionally, women need more privacy during visiting hours and better pain management than men, so that they can feel comfortable in the hospital (Teunissen et al., 2016). Moreover, the results of Elliott et al. show similar outcomes from the US. Female participants evaluated the hospital experience as less positive than males, especially for communication about medicines, discharge information, and cleanliness (Elliott et al., 2012). The studies reveal initial findings that humans of different genders and personalized attributes perceive care differently and this can have an impact on recovery.

**Fig. 1:**
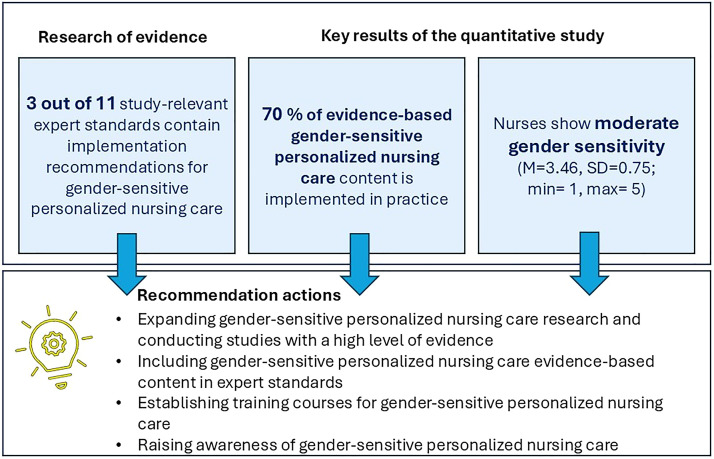
Key results of the HeartGap-study with derived recommendation actions from the HeartGap project.

The mean value index of the gender-sensitive personalized nursing care implementation items indicates that gender-sensitive personalized nursing care is rated highly in hospitals by nurses (M = 0.70). Less considered aspects of implementation in practice regarding the index include the recording of ethnicity, gender-sensitive aids for incontinence, and the staffing of shifts with both female and male nursing professionals (if possible). The latter aspect is based on the large proportion of female nurses compared to male nurses (82% female vs. 18% male (Radtke, 2024)), as well as on shift planning that takes into account compatibility and effective collaboration among nursing staff (Mollenhauer et al., 2026). There is potential for improvement and increased awareness in gender- and culturally sensitive hospital structures. Fig. 1 shows key results of the study with derived recommendation actions.

The results of the multiple linear regression show that predictors of hospital size are related to the degree of implementation. Teaching/research (university) hospitals and hospitals with >800 beds have a significantly negative relation to the degree of implementation in comparison to hospitals with ≤800 beds. Personal-related attributes have not become significant. The result can be interpreted as follows: The implementation of gender-sensitive personalized nursing care is not associated with individual nurse characteristics but rather with hospital structures and guidelines. This finding may help identify suitable implementation strategies. The literature review by Mollenhauer et al. identified several implementation measures that could support organizational structures (e.g., hospital structures) in becoming more gender sensitive (Mollenhauer et al., 2025). These are, for example, the implementation of a diversity commissioner (Erdsiek et al., 2022), internal training programs and quality circle for gender-sensitive personalized nursing care enhancing (Erdsiek et al., 2022; Kassenärztliche Bundesvereinigung, 2015), the integration of the two-step method with two options “current identity” and “sex assigned at birth”, or ethnicity in patient records (Deutsch et al., 2013; Lindsay and Kolne, 2020), the implementation of change agents in hospitals (Grasso et al., 2019), and achieving gender balance among employees and in leading positions (Binder-Fritz and Rieder, 2014). In this context and under current conditions, gender balance implies that hospital HR managers should ensure (where possible) that at least one male nurse is scheduled per shift, so that male patients have the option of receiving same-sex nursing care. Furthermore, women still constitute the majority of nursing leaders; However, men are overrepresented relative to their overall proportion in the nursing workforce, which is predominantly female. This imbalance becomes more pronounced at higher managerial levels, such as nursing directorates (Pflege-Netzwerk Deutschland, 2022).

Moreover, the results of the multiple linear regression analysis indicate that the predictor, country of birth, is associated with the degree of gender sensitivity. Nurses born in Germany appear to be more gender-sensitive than those born in other countries. The reasons for this result need further investigation. Structure-related attributes have not become significant. One possible explanation is that individuals with a migration background may have experienced greater difficulty completing the N-GAMS due to language barriers compared to those without a migration background. The understanding problems after the translation of the N-GAMS into the German language are known from other studies (Steinböck et al., 2015; Wortmann et al., 2023). In addition, the results could be explained by a person’s character, cultural background, and cultural interpretation of questionnaire items, training, and area of responsibility. In general, for qualifications and professional degrees obtained abroad, their equivalence must be recognized in Germany. This is challenging because immigrant nursing staff have often completed academic, medical-related training that differs significantly from nursing education in Germany. The nursing education program in Germany is highly generalist, grounded in evidence-based nursing science, and focuses on independence, preventive care, care planning, and documentation. Immigrant nursing staff are often required to complete continuing education programs, adaptation courses, or examinations to achieve full recognition of their qualifications in Germany and to clearly delineate their professional area of responsibility (Ritter, 2023). It should also be noted that there are statistical uncertainties and limitations in the study that may influence statistical variations measurement non-equivalence, subgroup imbalance, or residual confounding.

The correlation analysis showed that nurses with higher gender sensitivity tend to rate gender-sensitive personalized nursing care implementation less favorably. In a previous qualitative study as part of the HeartGap project involving interviews with nurses on the topic of gender-sensitive personalized nursing care, it became evident that many participants were challenged to associate meanings with the term gender-sensitive personalized nursing care when asked for a definition (Mollenhauer et al., 2026). Sensitized nurses may assess the implementation more critically because they are more aware of the relevant factors to consider.

Establishing a framework for gender-sensitive personalized nursing care in practice, the mandatory inclusion of gender-sensitive content from studies in the expert standards would be a supportive measure for nationwide implementation. Currently, only 3 of the 12 expert standards contain measures for implementation. The methods paper for the development of the expert standards should explicitly refer to the consideration of gender-sensitive personalized nursing care (Deutsches Netzwerk für Qualitätsentwicklung in der Pflege, 2025b).

In Germany, gender-sensitive care is anchored in law (§ 2b of the German Social Security Code V). Nevertheless, implementation is lagging behind the requirements. The current government would like to promote gender- and diversity-sensitive care, particularly in prevention, therapy, and research (Christlich Demokratische Union Deutschlands (CDU), Christlich-Soziale Union in Bayern e.V. (CSU), Sozialdemokratische Partei Deutschlands, 2025). As the self-governance in the healthcare system is not fulfilling the mandate of the legal framework, further compulsory laws to establish gender-sensitive personalized nursing care in practice would be supportive. This includes, for example, gender-sensitive personalized nursing care integration into training and academic curricula, continuing education opportunities in gender-sensitive personalized nursing care, and gender- and person-sensitive hospital and healthcare structures.

### 4.1 Limitations

A cross-sectional analysis was planned. However, the data collection took nine months, which means that external factors such as political decisions or further training opportunities could have biased the status quo survey. The survey period had to be extended because the response rate was lower than expected, despite the reminder strategy.

Due to the anonymous character of the survey, it is not possible to transparently state how many of the 994 hospitals responded, and how many nursing staff from each participated in the survey. Furthermore, given the voluntary character of participation in the study, self-selection and non-response bias must be considered as limiting factors. The findings may be influenced by the fact that nurses with greater gender sensitivity and interest in the topic were more likely to participate in the study.

The sample based on personal characteristics is approximately representative. The surveyed sample is slightly younger (39,6 vs. 40,6) and more academically qualified (17,7% vs. 0,5%) than the German average for nurses (Deutscher Bundestag, 2020; Rottenkolber and Thiele, 2025). As the collected sample does not comply with the a priori calculation regarding region and size of hospitals, weights per stratum were used. The weighting is intended to generate representative data. Nevertheless, biases may have resulted from the weighting in the statistical evaluation. Moreover, it should be noted that the regressions included 100 and 107 participants, respectively. Given the relatively small sample size, some uncertainty (e.g., possibility of overfitting, unstable coefficients, type II error, and uncertainty around statistically significant) regarding the interpretation of the results cannot be ruled out. Nevertheless, the statistical analysis provides initial valuable insights into gender-sensitive personalized nursing care and its potential correlations. Further detailed research needs to be conducted.

The results are based on a questionnaire survey with nurses from cardiology wards. The transferability of the study results to other specialties may be limited. Since the cardiology department has been addressing gender-specific issues for a longer time than other departments, the implementation rate of gender-sensitive personalized nursing care and the gender sensitivity of nursing staff in other departments may be lower.

## 5 Conclusion

Gender-sensitive personalized nursing care is part of patient-centered care and is important to improve evidence-based, high-quality care. Appropriate organizational structures and sensitized nurses are required for practical implementation in institutions of care. The findings of this study indicate that nurses demonstrate a moderate level of gender sensitivity, despite the absence of prior targeted training in gender-sensitive personalized nursing care. Nursing education evidently incorporates person-centeredness to such an extent that nurses intuitively apply gender-sensitive personalized nursing care principles in their daily practice (Mollenhauer et al., 2026). Academic and vocational training in gender-sensitive personalized nursing care is currently lacking in the curriculum (Seeland et al., 2020). Overall, academic training should be promoted to improve the quality of complex patient care.

Nurses assessed the institutional structures as moderately to highly gender sensitive. This includes, for example, patient-centered admission management and menu selections, shift schedules involving both female and male nurses, and gender-sensitive incontinence management for patients concerned. Only 3 of the 12 expert standards contain measures for implementation, and this is insufficient for a holistic, gender-sensitive approach to patient care. The potential for further development can be increased through the following measures. The methods paper for the development of expert standards should explicitly recommend that experts consider gender-sensitive personalized nursing care when developing expert standards. These standards are the foundation for hospital internal standard operating procedures and offer direct access to healthcare providers. Additionally, compulsory training in implementing gender-sensitive personalized nursing care is required to support knowledge expansion. The system of mandatory annual continuing education for physicians, based on the accumulation of credit points, could be adapted for nursing professionals. It could enhance and standardize nursing care quality. Given that continuing education requirements are administered by professional associations and that nursing chambers are not uniformly established across all German federal states, implementing this concept would currently require diverse administrative frameworks.

Especially in hospital settings, nurses provide more intensive time with patient care compared to physicians, which enables them to gain a more comprehensive understanding of their patients regarding pain status, life quality, or family status. Gender-sensitive symptoms of myocardial infarction or high blood pressure can be quickly and correctly assessed by nursing professionals, which can avert undesirable outcomes through interdisciplinary care. Moreover, nurses can become multipliers, disseminating knowledge about gender-specific symptoms to patients and increasing health literacy by empowering individuals to recognize symptoms early, communicating actively and effectively with healthcare professionals, and making informed decisions.

## Data

Since nurses were assured upon participation that the data would not be shared, the data cannot be published in a repository.

## Declaration of generative AI use

AI was not used for data collection or manuscript preparation.

## Funding

The German Innovation Fund funds the project with the funding number: 01VSF22030.

## CRediT authorship contribution statement

**Judith Mollenhauer:** Writing – original draft, Methodology, Data curation, Conceptualization. **Sophia Sgraja:** Writing – review & editing, Supervision, Methodology, Conceptualization. **Martina Kloepfer:** Writing – review & editing, Supervision, Methodology, Conceptualization. **Ute Seeland:** Writing – review & editing, Supervision, Methodology, Conceptualization. **Volker E. Amelung:** Writing – review & editing, Supervision, Methodology, Conceptualization. **Clarissa Kurscheid:** Writing – review & editing, Supervision, Methodology, Conceptualization.

## Declaration of competing interest

The authors declare that they have no known competing financial interests or personal relationships that could have appeared to influence the work reported in this paper.

The author is an Editorial Board Member/Editor-in-Chief/Associate Editor/Guest Editor for this journal and was not involved in the editorial review or the decision to publish this article.

The authors declare the following financial interests/personal relationships which may be considered as potential competing interests

## Appendix Supplementary materials

Image, application 1

Image, application 2

Image, application 3

## References

[bib0001] Aliri J., Prego-Jimenez S., Goñi-Balentziaga O., Pereda-Pereda E., Perez-Tejada J., Labaka Etxeberria A. (2022). Gender awareness is also nurses' business: measuring sensitivity and role ideology towards patients. J. Nurs. Manage.

[bib0002] American Association of Colleges of Nursing (2024). Nursing workforce fact Sheet. https://www.aacnnursing.org/news-data/fact-sheets/nursing-workforce-fact-sheet.

[bib0003] Besharati S., Kalaleh A.R. (2024). Personalized nursing and precision nursing: a concept of the future of the health model. J. Prev. Diagn. Treat. Strateg. Med..

[bib0004] Binder-Fritz C., Rieder A. (2014). Zur verflechtung von Geschlecht, sozioökonomischem status und ethnizität im kontext von Gesundheit und Migration. Bundesgesundheitsblatt. Gesundheitsforschung. Gesundheitsschutz..

[bib0005] Bundesinstitut für Berufsbildung (2024). Rahmenausbildungspläne der Fachkommission nach § 53 PflBG. https://www.bibb.de/dienst/publikationen/de/20123.

[bib0006] Christlich Demokratische Union Deutschlands (CDU) (2025). Christlich-Soziale Union in Bayern e.V. (CSU), sozialdemokratische Partei Deutschlands. Verantwortung für Deutschland: koalitionsvertrag zwischen CDU, CSU und SPD 21. Legislaturperiode. https://www.koalitionsvertrag2025.de/.

[bib0007] Destatis. Grunddaten der Krankenhäuser (2022). https://www.destatis.de/DE/Themen/Gesellschaft-Umwelt/Gesundheit/Krankenhauser/Publikationen/Downloads-Krankenhaeuser/grunddaten-krankenhaeuser-2120611217004.pdf?__blob=publicationFile&v=6.

[bib0008] Deutsch M.B, Green J., Keatley J., Mayer G., Hastings J., Hall A.M. (2013). Electronic medical records and the transgender patient: recommendations from the world professional association for transgender health emr working group. J. Am. Med. Inform. Assoc. JAMIA.

[bib0009] Deutsche Gesellschaft für Geschlechtsspezifische Medizin (DGesGM) e.V (2025). Willkommen bei der Deutschen Gesellschaft für geschlechtsspezifische medizin!. https://dgesgm.de/de/.

[bib0010] Deutscher Berufsverband für Pflegeberufe (2026). Unsere Inhouse-Schulungen – Ihr Schlüssel zu nachhaltigem erfolg. https://www.dbfk.de/de/dbfk/nordwest/bildung/inhouse.php.

[bib0011] Deutscher Bundestag (2020). Hohes Durchschnittalter der Azubis in Alten- und Krankenpflege. https://www.bundestag.de/webarchiv/presse/hib/2020_09/710768-710768.

[bib0012] Deutsches Netzwerk für Qualitätsentwicklung in der Pflege (2025). Deutsches Netzwerk für Qualitätsentwicklung in der Pflege Expertenstandards und Auditinstrumente. https://www.dnqp.de/expertenstandards-und-auditinstrumente/.

[bib0013] Deutsches Netzwerk für Qualitätsentwicklung in der Pflege (2025). Methodisches vorgehen zur entwicklung und aktualisierung von expertenstandards in der Pflege. https://www.dnqp.de/fileadmin/HSOS/Homepages/DNQP/Dateien/Weitere/DNQP-Methodenpapier2025.pdf.

[bib0014] Elliott M.N, Lehrman W.G, Beckett M.K, Goldstein E., Hambarsoomian K., Giordano L.A. (2012). Gender differences in patients' perceptions of inpatient care. Health Serv. Res..

[bib0015] Erdsiek F., Aksakal T., Mader M., Idris M., Yılmaz-Aslan Y., Razum O. (2022). Diversity-sensitive measures in German hospitals - attitudes, implementation, and barriers according to administration managers. BMC. Health Serv. Res..

[bib0016] Gattino S., Molinengo G., de P.N. (2024). Primary care physicians and gender medicine: validation of the italian version of nijmegen gender awareness in medicine scale (N-GAMS). TPM.

[bib0017] German Science And Humanities Council (2023). Perspektiven für die weiterentwicklung der gesundheitsfachberufe | wissenschaftliche potenziale für die gesundheitsversorgung erkennen und nutzen. https://www.wissenschaftsrat.de/download/2023/1548-23.

[bib0018] Goussault-Capmas P., Panjo H., Pelletier-Fleury N. (2024). Gender awareness among general practitioners in France: a cross sectional study using the nijmegen gender awareness in medicine scale (N-GAMS). Sci. Rep..

[bib0019] Grasso C., McDowell M.J, Goldhammer H., Keuroghlian A.S. (2019). Planning and implementing sexual orientation and gender identity data collection in electronic health records. J. Am. Med. Inform. Assoc. JAMIA.

[bib0020] Jandaghian-Bidgoli M., Jamalnia S., Pashmforosh M., Shaterian N., Darabiyan P., Rafi A. (2025). Personalized nursing as the missing link of providing care: a systematic review. BMC. Nurs..

[bib0021] Kassenärztliche Bundesvereinigung (2015). Aspekte einer geschlechtersensiblen Gesundheitsversorgung: handbuch Qualitätszirkel. https://www.kbv.de/media/sp/4.19_Geschlechtersensible_Gesundheitsversorgung.pdf.

[bib0022] Kassenärztliche Bundesvereinigung (2023). Einrichtungen und Betten. https://www.kbv.de/infothek/zahlen-und-fakten/gesundheitsdaten/krankenhaeuser-und-betten.

[bib0023] Kiesl H., Baur N., Blasius J. (2022). Handbuch Methoden der Empirischen Sozialforschung.

[bib0024] Labaka A., Zamakola A., Arrue M., Arrieta H. (2023). Evaluating gender awareness, gender-related health knowledge and patient pain legitimation among nursing students: a quasi-experimental study. Nurse Educ. Pract..

[bib0025] Legato M.J, Colman C. (1991).

[bib0026] Lindsay S., Kolne K. (2020). The training needs for gender-sensitive care in a pediatric rehabilitation hospital: a qualitative study. BMC. Med. Educ..

[bib0027] Lopes-Júnior L.C. (2021). Personalized nursing care in precision-medicine era. SAGe Open. Nurs..

[bib0028] Mollenhauer J., Sgraja S., Seeland U., Kloepfer M., Amelung V.E, Kurscheid C. (2025). Multifarious approaches of implementation to transfer gender sensitivity in health care practice: a scoping review. BMC. Health Serv. Res..

[bib0029] Mollenhauer J., Sgraja S., Seeland U., Kloepfer M., Amelung V.E, Kurscheid C. (2026). Implementing gender-sensitive personalized nursing care into practice - a qualitative study with nurses from the cardiology units. BMC. Nurs..

[bib0030] Pflege-Netzwerk Deutschland (2022). Wie mehr frauen in führungspositionen kommen. https://pflegenetzwerk-deutschland.de/wie-mehr-frauen-in-fuehrungspositionen-kommen.

[bib0031] Prego-Jimenez S., Pereda-Pereda E., Perez-Tejada J., Aliri J., Goñi-Balentziaga O., Labaka A. (2022). The impact of sexism and gender stereotypes on the legitimization of women's low back pain. Pain Manag. Nurs. Off. J. Am. Soc. Pain Manag. Nurses.

[bib0032] Radtke R. (2024). Geschlechterverteilung unter sozialversicherungspflichtig beschäftigten in der Pflege und insgesamt in Deutschland im Jahr 2023. https://de.statista.com/statistik/daten/studie/1029877/umfrage/verteilung-von-pflegekraefte-in-deutschland-nach-pflegeart-und-geschlecht/.

[bib0033] Regitz-Zagrosek V., Oertelt-Prigione S., Regitz-Zagrosek V. (2012). Sex and Gender Aspects in Clinical Medicine.

[bib0034] Ritter M. (2023). https://www.bpb.de/themen/migration-integration/regionalprofile/deutschland/543561/herausforderungen-fuer-zugewanderte-pflegekraefte/.

[bib0035] Rottenkolber D., Thiele G. (2025). Akademisierte Pflegefachkräfte: berufsverbleib?. Pflegezeitschrift.

[bib0036] Runkler T.A. (2025).

[bib0037] Seeland U., Dettmer S., Ludwig S., Kaczmarczyk G., Kohl R., Kühn K. (2020). Aktueller Stand der integration von Aspekten der Geschlechtersensibilität und des Geschlechterwissens in Rahmenlehr- und Ausbildungsrahmenpläne, Ausbildungskonzepte, -curricula und Lernzielkataloge für beschäftigte im Gesundheitswesen. https://www.aerztinnenbund.de/downloads/7/Gutachten_Integration_von_Aspekten_der_Geschlechtersensibilitaet.pdf.

[bib0038] Sgraja S., Mollenhauer J., Kloepfer M., Seeland U., Kurscheid C., Amelung V. (2024). Gender health gaps in guideline-based inpatient cardiovascular medical and nursing care and implementation strategies to reduce the gap (HeartGap): a mixed methods study protocol. PLoS. One.

[bib0039] Shamasneh B., Nemer M., NME A.-R. (2023). Gender awareness in healthcare: contextualization of an arabic version of the nijmegen gender awareness in medicine scale (N-GAMS). Healthcare (Basel).

[bib0040] Steinböck S., Lydtin S., Hofhansl A., Kautzky-Willer A. (2015). Gender awareness bei medizinstudierenden der Medizinischen Universität Wien. Eine empirische analyse von geschlechtersensibilität und geschlechterstereotypisierungen. FZG/FGS.

[bib0041] Teunissen T.AM., Rotink M.E, Lagro-Janssen A.LM. (2016). Gender differences in quality of care experiences during hospital stay: a contribution to patient-centered healthcare for both men and women. Patient. Educ. Couns..

[bib0042] van Servellen G. (1988). Nurses' perceptions of individualized care in nursing practice. West J. Nurs. Res..

[bib0043] Verdonk P., Benschop Y.W.M., de H.H., Lagro-Janssen T.LM. (2008). Med. Stud. Gend. Aware., Sex Roles.

[bib0044] World Health Organization (2025). Gender and health. https://www.who.int/health-topics/gender#tab=tab_1.

[bib0045] Wortmann L., Haarmann L., Yeboah A., Kalbe E. (2023). Gender medicine teaching increases medical students' gender awareness: results of a quantitative survey. GMS. J. Med. Educ..

[bib0046] Yang M., Ta N., Bai X., Wei C., Sun C., Han C. (2023). The effectiveness of personalized nursing on quality of life in cardiovascular disease patients: a systematic review and meta-analysis. Evid.-Based Complement. Altern. Med. eCAM.

