# Supplementary material for: How gender-sensitive is nursing care in hospitals? Results of a national questionnaire survey in cardiology in Germany
Source: Int J Nurs Stud Adv. 2026 Jul 15;11:100631. doi: 10.1016/j.ijnsa.2026.100631 (PMC13427392; doi:10.1016/j.ijnsa.2026.100631)
Supplement: Supplementary file 1 [file mmc1.pdf]

Gefördert durch:
Gemeinsamer Bundesausschuss
Innovationsausschuss

HeartGap
geschlechterspezifische Versorgung

Gender Aspects in Nursing Expert Standards

Authors: Judith Mollenhauer[a], Luca Mehlig[a], Sophia Sgraja[b], Prof. Dr. Volker E. Amelung[b], Prof. Dr. Clarissa Kurscheid[a]

[a] figus GmbH – Private Research Institute for Health and System Design
[b] Institute for Epidemiology, Social Medicine and Health Systems Research, Hannover Medical School

The *HeartGap* project (funding code 01VSF22030) is a health services research study funded by the Innovation Fund, running from January 1, 2023, to June 30, 2025, with a funding volume of €604,000. The consortium consists of the private research institute for health and system design (figus GmbH) and Hannover Medical School. The project is supported in terms of content and communication by the Institute for Gender Health e.V. with Dr. Martina Kloepfer and Prof. Dr. Ute Seeland (Chair of Internal Medicine/Gender-Sensitive Medicine at Otto von Guericke University Magdeburg).

The acronym *HeartGap* stands for *Gender Health Gaps in guideline-based inpatient cardiological care and implementation strategies to reduce them*. The project investigates the extent to which evidence-based, gender-sensitive, and personalized nursing and medical care are implemented in clinical cardiology settings and identifies patients' needs.

Overview

The gender of both patients and nursing professionals can play a role in various aspects of care delivery and communication. Gender-sensitive care represents an important step toward personalized nursing.

Gender is defined by both biological sex (female, male, intersex) and gender as a social construct (roles, attributes, and behaviors defined by society). In addition, other diversity factors—such as age, migration background, religious affiliation, sexual orientation, and physical and psychological characteristics—must be considered as individual patient characteristics in nursing care.

This summary provides an overview of relevant knowledge and implementation measures for gender-sensitive nursing. It is based on a systematic review of the expert standards of the German Network for Quality Development in Nursing (DNQP), using gender-specific keywords (gender, woman/women, man/men, female, male) as well as additional diversity-related factors.

The content of the expert standards was developed based on systematic literature reviews and expert knowledge. Due to the underlying evidence base, the standards primarily reflect a binary understanding of gender.

The following table summarizes gender- and diversity-sensitive content as well as explicit measures for clinical practice across the expert standards considered (e.g., chronic wounds, discharge management, pain management, fall prevention, dementia care, decubitus prevention, urinary continence promotion, nutrition management, oral health, skin integrity, and mobility promotion).

Expertstandards	Gender aspects
**Chronic wounds**[1]	Patients with diabetes may experience difficulties in foot self-care due to:<br>• lack of information<br>• lack of support systems and role models<br>• health-related barriers (e.g., visual impairment, obesity)<br>• fear of making mistakes due to comorbidities<br>• influences of age, gender, and educational level<br>Patients with diabetic foot ulcers or pressure ulcers report changes in body image, particularly women.
**Discharge management**[2]	Predictors influencing post-discharge care in stroke patients include age, gender, diabetes mellitus, heart disease, stroke history, snoring, and caregiving relatives.<br>The increasing number of older people living alone—particularly women—requires adapted care concepts.<br>**Measures:**<br>• Gender- and culturally sensitive aspects should be considered in patient and family education.
**Pain management**[3]	**General:**<br>Girls and women report a significantly higher frequency of pain.<br>**Joint pain:**<br>Women (29.3%) are more likely to experience joint pain than men (24.4%).<br>**Chronic pain:**<br>Women experience chronic pain more frequently than men. These differences tend to converge up to the age of 80, with the highest prevalence observed among women over 65 years of age. Women generally exhibit lower pain thresholds and pain tolerance and are therefore likely to perceive pain as more intense and more distressing. The underlying mechanisms are not yet fully understood; however, existing research suggests hormonal (e.g., estrogen-related) and genetic factors.

	**Chronic pain and disability:**<br>Chronic pain is common among individuals with intellectual and/or physical disabilities, particularly among women living in independent or community-based settings.
**Fall prevention**[4]	**Androgen receptors:**<br>The use of androgen receptor inhibitors in the treatment of prostate cancer in men is associated with an increased risk of falls.<br><br>Women aged 70 years and older (60% experienced a fall within the past 12 months) fall significantly more frequently than men (50%).<br><br>In long-term inpatient care settings, men (2.2 falls per resident per year) experience falls more frequently than women (1.5 falls).<br><br>**Measures**<br><br>**Osteoporosis:** Clinical guidelines recommend vitamin D supplementation for older women with osteoporosis. In addition, screening for osteoporosis is recommended for women aged 65 years or younger who are at increased risk of falls.
**Dementia care**[5]	The patient's sex does not influence staff–patient social interactions in nursing homes. Instead, factors such as the severity of cognitive impairment, level of dependency, and sexually aggressive behavior have a greater impact.<br><br>Women appear to respond more positively to stimuli such as dolls or babies than men.
**Decubitus prevention**[6]	Sex has been identified as a risk factor for the prevalence of pressure ulcers in several studies; however, the evidence supporting this association is limited and inconsistent.
**Urinary contience promotion**[7]	Urinary incontinence predominantly affects women and older individuals of all sexes.<br>In younger and middle-aged women, stress urinary incontinence is most common, whereas urge and mixed incontinence become more prevalent with increasing age.<br><br>Risk factors for urinary incontinence in women include pelvic floor strain (e.g., due to pregnancy and childbirth), obesity, positional changes or enlargement of the uterus, sexualized violence resulting in pelvic floor or vaginal injuries, and diabetes mellitus. A sensitive approach is essential when addressing experiences of sexualized violence during medical history taking.<br><br>Risk factors for urinary incontinence in men primarily include prostate disorders and prostate surgery.

Constipation (defined as fewer than two bowel movements per week) and defecation problems beginning in adolescence increase the risk of pelvic organ prolapse and urinary incontinence in women.

Treatment

Physical activity: Excessive physical activity may place strain on the pelvic floor in women, whereas moderate physical activity appears to have a protective effect and may reduce stress urinary incontinence, though not urge or mixed incontinence. However, the evidence base is limited, and the interpretation of existing data remains controversial. For men, no clear association between physical activity and urinary incontinence has been established.

Diagnostics: Measurement of post-void residual urine is particularly recommended for men who report a sensation of incomplete bladder emptying.

Pelvic floor training: Daily pelvic floor training is an effective intervention for the prevention of pregnancy-related urinary incontinence and for the treatment of women with stress and mixed incontinence. For men following prostatectomy, study findings are inconsistent.

Prior to initiating pelvic floor training, an assessment of pelvic floor muscle function should be performed. This may be conducted via digital examination—anally in men and vaginally in women—to evaluate muscle strength.

Biofeedback: Biofeedback and feedback interventions enhance the effectiveness of pelvic floor training in women with urinary incontinence.

Electrical stimulation: There is currently no evidence to suggest that electrical stimulation combined with pelvic floor training is more effective than pelvic floor training alone. Expert recommendations support the use of electrical stimulation in women who are unable to voluntarily contract their pelvic floor muscles. Subsequently, these patients should transition to active pelvic floor training. Electrical stimulation should not be used routinely in the treatment of women with overactive bladder.

Vaginal cones: In women with stress urinary incontinence, vaginal cones are preferable to no treatment. They may have a comparable effect to other therapeutic approaches, such as electrical stimulation or pelvic floor training. However, pelvic floor training appears to be more effective than vaginal cones in women with stress and mixed incontinence.

Bladder training: As bladder training is not associated with adverse effects, it is recommended as an early conservative intervention, particularly for women with urge urinary incontinence, before other or more invasive treatment options are considered. For women with stress and mixed incontinence, a combined approach of bladder training and pelvic floor training is more effective

than pelvic floor training alone. Due to the limited availability of data, no definitive conclusions can be drawn regarding the effectiveness of bladder training in men.

Management and Aids

To preserve privacy during toileting, it may be important for patients to be assisted by a nurse of the same sex.

Healthcare institutions should provide sex-specific toileting and incontinence aids and ensure that nursing staff are adequately trained in their use.

Intravaginal or intraurethral devices: Experts recommend that intravaginal or intraurethral devices for women should only be used in specific situations and not as part of routine care.

Silicone catheters may reduce adverse effects in the urethra, particularly in men.

Condom catheters: For men without increased post-void residual volume or significant voiding dysfunction, the use of a condom catheter is a suitable option.

Absorbent Products

While women are generally more familiar with absorbent materials due to menstrual hygiene, men often experience difficulties using these products and use them less frequently.

- Disposable incontinence pads are more effective for women with mild urinary incontinence than alternative products, such as menstrual hygiene products or washable pads.

For men with moderate to severe urinary incontinence, choosing appropriate products can be challenging; however, many prefer all-in-one systems over pads. For women, so-called pull-ups (pants) have been shown to be more advantageous than other products, although they are more expensive. Women living in community settings tend to prefer pull-ups, including for nighttime use.

Washable products (evaluated only in non-institutional settings) are rarely accepted by women, whereas some men find them acceptable for nighttime use.

Women who are mobile and independent tend to prefer smaller pads, regardless of the severity of incontinence.

**Nutrition management**[8]	Malnutrition: Female sex is one of several contributing factors to the development of malnutrition.<br>**Measures**<br>Healthcare institutions should provide meals that are tailored to patients' individual biographical, religious, and cultural backgrounds and should align eating practices and rituals with patients' personal habits and preferences.
**Oral health**[9]	Oral health: No sex-specific differences have been identified in relation to oral health.<br>However, oral health is of particular importance during pregnancy. Dental check-ups should be conducted both before and after childbirth.<br>**Measures**<br>The use of a neutralizing mouth rinse is recommended after episodes of vomiting.
**Skin integrity**[10]	Intertrigo: Female sex is one of several risk factors associated with the prevalence of intertrigo. The most commonly affected areas include the skin under the breasts (in women), the inguinal region, the abdomen, and the gluteal cleft.<br>**Measures**<br>Prevention of intertrigo: Direct skin-to-skin contact should be avoided. This can be achieved by wearing close-fitting, but not overly tight, underwear (e.g., with longer leg sections). The use of thong underwear is not recommended. Bras should preferably be made of cotton; if necessary, a cotton garment may be worn underneath the bra. In men, the use of a suspensory support may be beneficial.<br>Skin cleansing and care: Consideration of patient preferences regarding the sex of the caregiver is important during personal hygiene and skin care, in addition to maintaining dignity and privacy, ensuring meaningful communication, and fostering a respectful therapeutic relationship.
**Mobility promotion**[11]	No sex-specific risks or measures have been identified.

Key considerations for cardiology

Important additional sex-specific aspects in cardiology (not derived from DNQP expert standards)

- **Differences in myocardial infarction symptoms:**
  The typical severe chest pain radiating to various parts of the body occurs less frequently in women than in men. Other symptoms to consider include shortness of breath, unusual fatigue, prolonged symptoms (even at rest), pain in the back and abdomen, neck or jaw pain, and a strong, burning pressure in the chest. In women, the risk of myocardial infarction increases markedly during perimenopause.[12]
- **Differences in blood pressure values:**
  A blood pressure of 120/80 mmHg is commonly used as a general reference for optimal levels. However, blood pressure varies by sex and age. At younger ages, women generally have lower blood pressure than men; during perimenopause, blood pressure in women typically increases substantially and may exceed average values observed in men. Currently, guideline-based reference values stratified by sex and age are not available. The following table presents study-based average values according to age and sex.[13, 14]

**age**	**20-40 years**		**40-60 years**		**> 60 years**	
	**women**	**men**	**women**	**men**	**women**	**men**
**blood pressure (mmHg)**	105/68 bis 115/77	115/75 bis 120/82	115/77 bis 130/80	120/82 bis 130/84	130/68 bis 145/78	130/70 bis 140/82

Reference: Ji et al. (2024), figure 1.[15]

[1] Andreas Büscher et al. (2015), *Expertenstandard Pflege von Menschen mit chronischen Wunden*, 1. Aktualisierung, Deutsches Netzwerk für Qualitätsentwicklung in der Pflege (Hrsg.). Hochschule Osnabrück.

[2] Bärbel Dangel et al. (2019*), Expertenstandard Entlassungsmanagement in der Pflege*, 2. Aktualisierung, Deutsches Netzwerk für Qualitätsentwicklung in der Pflege (Hrsg.). Hochschule Osnabrück.

[3] Jürgen Osterbrink et al. (2020), *Expertenstandard Schmerzmanagement in der Pflege*, Aktualisierung 2020, Deutsches Netzwerk für Qualitätsentwicklung in der Pflege (Hrsg.). Hochschule Osnabrück.

[4] Michael Simon et al., *Expertenstandard Sturzprophylaxe in der Pflege*, 2. Aktualisierung, Deutsches Netzwerk für Qualitätsentwicklung in der Pflege (Hrsg.). Hochschule Osnabrück.

[5] Martina Roes et al. (2019), *Expertenstandard Beziehungsgestaltung in der Pflege von Menschen mit Demenz*, Deutsches Netzwerk für Qualitätsentwicklung in der Pflege (Hrsg.). Hochschule Osnabrück.

[6] Jan Kottner et al. (2017), *Expertenstandard Dekubitusprophylaxe in der Pflege*, 2. Aktualisierung (Hochschule Osnabrück, 2017).

[7] Andreas Büscher et al. (2014), *Expertenstandard Förderung der Harnkontinenz in der Pflege*, 1. Aktualisierung, Deutsches Netzwerk für Qualitätsentwicklung in der Pflege (Hrsg.). Hochschule Osnabrück.

[8] Sabine Bartholomeyczik et al. (2017), *Expertenstandard Ernährungsmanagement zur Sicherung und Förderung der oralen Ernährung in der Pflege*, 1. Aktualisierung, Deutsches Netzwerk für Qualitätsentwicklung in der Pflege (Hrsg.). Hochschule Osnabrück.

[9] Erika Sirsch et al. (2021), *Expertenstandard Förderung der Mundgesundheit in der Pflege*, Deutsches Netzwerk für Qualitätsentwicklung in der Pflege (Hrsg.). Hochschule Osnabrück.

[10] Jan Kottner et al. (2023), *Expertenstandard Erhaltung und Förderung der Hautintegrität in der Pflege*, Deutsches Netzwerk für Qualitätsentwicklung in der Pflege (Hrsg.). Hochschule Osnabrück.

[11] Klaus Wingenfeld et al. (2020*), Expertenstandard Erhaltung und Förderung der Mobilität in der Pflege*, 1. Aktualisierung, Deutsches Netzwerk für Qualitätsentwicklung in der Pflege (Hrsg.). Hochschule Osnabrück.

[12] Ute Seeland (2023), *Geschlechtersensible medizinische Ansätze in der Kardiologie*, [Gender-sensitive medical approaches in cardiology] Dtsch Med Wochenschr 148, Nr. 09: 539, 541, https://doi.org/10.1055/a-1892-4687.

[13] Andrea Baessler et al. (2024), *Geschlechterspezifische Aspekte kardiovaskulärer Erkrankungen*, Die Kardiologie 18, Nr. 4: 294, https://doi.org/10.1007/s12181-024-00694-9.

[14] Hongwei Ji et al. (2020), *Sex Differences in Blood Pressure Trajectories Over the Life Course*, JAMA Cardiology 5, Nr. 3, https://doi.org/10.1001/jamacardio.2019.5306.
