# Supplementary material for: How gender-sensitive is nursing care in hospitals? Results of a national questionnaire survey in cardiology in Germany
Source: Int J Nurs Stud Adv. 2026 Jul 15;11:100631. doi: 10.1016/j.ijnsa.2026.100631 (PMC13427392; doi:10.1016/j.ijnsa.2026.100631)
Supplement: Supplementary file 2 [file mmc2.pdf]

HeartGap questionnaire for nurses – English translation

Dear Participant,
Thank you for your willingness to participate in this survey.
As part of the HeartGap project funded by the Innovation Fund, we would like to ask you about your assessment and experiences regarding gender-sensitive care. The Hannover Medical School (MHH) and the Private Research Institute for Health and System Design (figus GmbH) have been commissioned by the Federal Joint Committee to conduct the evaluation.

The survey takes about 10 minutes. We assure you that all your responses will be anonymized, and no conclusions can be drawn about your identity. The findings will be used to assess the current state of gender-sensitive care. Data will not be shared with third parties. Anonymized data will be published in professional journals.

Please complete the questionnaire as fully as possible and follow your first instinct.
Thank you for your participation!

Questions?
Judith Mollenhauer, M.Sc. (Research Associate at figus Institute)
Email: j.mollenhauer@figus.koeln
Phone: +49 221 29257382

Part A: About the person

A1. Gender:
- Female
- Male
- Diverse

A2. Age (in years):
[Free text]

A3. Born in Germany?
- Yes
- No

A4. Highest school qualification:
- (Technical) High School Diploma or equivalent
- Secondary School Certificate or equivalent
- Lower Secondary School Certificate

- None
- Other: [Free text]

A5. Highest professional qualification:
- Doctorate
- (Technical) University Degree (Bachelor, Master)
- Completed vocational training
- No professional qualification
- Currently in training
- Other: [Free text]

A6. Working in nursing since [including training]... (in years):
[Free text]

A7. Size of the institution where you work:
- University hospital (>800 beds & teaching/research)
- Maximum care facility (>800 beds)
- Specialized care facility (501–800 beds)
- Basic care facility (≤500 beds)

A8. Region of the institution:
- Large city (≥100,000 inhabitants)
- Town (5,000–99,999 inhabitants)
- Rural community (<5,000 inhabitants)

Part B: Assessment of Gender Sensitivity

Please respond to the following statements.

B1. Statements on "Gender Differences in Nursing"
(Scale: Strongly disagree (--) to Strongly agree (++))

- Nurses´ knowledge of gender differences in illness and health increases quality of care.
- Nurses should only deal with biological differences between men and women.
- In non-sex-specific health disorders, the sex/ gender of the patient is irrelevant.
- A nurse should confine as much as possible to biomedical aspects of health complaints of men and women.
- Nurses do not need to know what happens in the lives of men and women to be able to deliver medical care.
- Differences between female and male nurses are too small to be relevant.
- Especially because men and women are different, nurses should treat everybody the same.
- Nurses who address gender differences are not dealing with the important issues.
- In communicating with patients, it does not matter to a nurse whether the patients are men or women.

- In communicating with patients, it does not matter the nurse is a man or a woman
- Differences between male and female patients are so small that nurses can hardly take them into account.
- For effective treatment, nurses should address gender differences in etiology and consequences of disease.
- It is not necessary to consider gender differences in presentation of complaints.

Part C: Questions on Gender-Sensitive Care

C1. Discharge Management Standard:
For inpatients, we always record...
(Options: Yes / No / Sometimes / Don't know)
- Age
- Ethnic background
- Gender
- Chronic illnesses

C2. Pain Management Standard:
Estrogens and female genetics can play a role in pain perception and prevalence.
(Yes / No)

C3. Fall Prevention Standard:
(Scale: Does not apply – Fully applies – Don't know)
- I initiate special fall prevention measures when a prostate cancer patient is taking androgen receptor inhibitors.

C4. Promotion of Urinary Continence Standard:
(Scale: Does not apply – Fully applies – Don't know)
- We consider the wish of patients with incontinence to be cared for by a same-gender nurse (if possible).
- We provide visual aids for counseling patients with incontinence.
- We have various incontinence aids available to allow for situational and gender-specific selection.
- Our facility ensures that all men's restrooms have large enough bins next to the toilet for disposing of incontinence pads and pants.
- We consider gender diversity in staffing so that both male and female nurses work together on a shift (if available).

C5. Urinary Continence Standard (continued):
(Scale: Does not apply – Fully applies – Don't know)
- For bedridden patients, we use toilet aids...
  - ...specifically designed for female anatomy.
  - ...specifically designed for male anatomy.

C6. Nutrition Management Standard:
(Scale: Does not apply – Fully applies – Don’t know)
- Our facility allows for consideration of influencing factors (e.g., biographical, religious, cultural) in patients’ nutrition and table manners.

C7. Gender Differences in Heart Attack Symptoms:
Women more often present with symptoms such as unusual fatigue, shortness of breath, and nausea during an acute myocardial infarction than men.
(Yes / No)

C8. Final Question:
(Scale: Very well – Well – Poorly – Not at all – Cannot judge)
- To what extent is gender-sensitive care implemented on your ward (e.g., respecting patient privacy, religious affiliation, space for prayer, consideration of migration background, language barriers, dietary habits)?

C9. Optional:
What role does the gender of the patient play in nurse-patient communication in your view?
[Free text]

C10. Additional Comments (optional):
[Free text]

Thank you very much for your participation!
If you are interested in a summary of the results from the final part of the survey on gender-sensitive care standards, please send an informal email to:
j.mollenhauer@figus.koeln
Phone: +49 221 29257382
Judith Mollenhauer, M.Sc. (Research Associate at figus Institute)
