# Supplementary material for: How gender-sensitive is nursing care in hospitals? Results of a national questionnaire survey in cardiology in Germany
Source: Int J Nurs Stud Adv. 2026 Jul 15;11:100631. doi: 10.1016/j.ijnsa.2026.100631 (PMC13427392; doi:10.1016/j.ijnsa.2026.100631)
Supplement: Supplementary file 3 [file mmc3.pdf]

LimeSurvey

**Liebe Teilnehmerin, lieber Teilnehmer,**

**vielen Dank für Ihre Bereitschaft, an der Befragung teilzunehmen!**

**Im Rahmen des Innovationsfonds geförderten Projekts HeartGap möchten wir Sie zu Ihrer Einschätzung und Erfahrungen zum Thema geschlechtersensible Pflege befragen.**

**Die Medizinische Hochschule Hannover (MHH) und das Priv. Forschungsinstitut für Gesundheits- und Systemgestaltung (figus GmbH) sind vom Gemeinsamen Bundesausschuss beauftragt worden, die Evaluation durchzuführen.**

**Die Befragung dauert etwa 10 Minuten. Wir versichern Ihnen, dass alle Ihre Angaben anonymisiert ausgewertet werden und kein Rückschluss auf Ihre Person möglich ist. Die Erkenntnisse werden dafür genutzt, einen Status quo über die geschlechtersensible Versorgung zu erheben. Das Weiterleiten der Daten an Dritte ist ausgeschlossen. Anonymisierte Daten werden in Fachpublikationen veröffentlicht.**

**Bitte füllen Sie den Fragebogen möglichst vollständig aus und lassen Sie sich von Ihrem ersten Gedanken leiten. Vielen Dank für Ihre Teilnahme!**

**Haben Sie Fragen?**

**Judith Mollenhauer M.Sc. (wissenschaftliche Mitarbeiterin des figus-Instituts)**

**E-Mail: j.mollenhauer@figus.koeln**

**Tel.: 0221-29257382**

# Teil A: Zur Person

Bitte beantworten Sie nachfolgend die Fragen zu Ihrer Person.

**A1. Geschlecht:**

weiblich ☐

männlich ☐

divers ☐

**A2. Alter (Angabe in Jahren):**

**A3. In Deutschland geboren?**

Ja ☐

Nein ☐

**A4. Höchster schulischer Abschluss:**

(Fach-)Gymnasium/(Fach-)Abitur/vglb. ☐

Mittlere Reife/Realschule/vglb. ☐

Hauptschulabschluss ☐

keiner ☐

Sonstiges ☐

Sonstiges

**A5. Höchster fachspezifischer, beruflicher Abschluss:**

- Promotion ☐
- (Fach-)Hochschulabschluss (Bachelor, Master) ☐
- Abgeschlossene Berufsausbildung ☐
- ohne beruflichen Bildungsabschluss ☐
- derzeit in Ausbildung ☐
- Sonstiges ☐

Sonstiges

**A6. Im pflegerischen Beruf tätig seit [inkl. Ausbildung]... (Angabe in Jahren)**

**A7. Größe der Einrichtung, in der Sie arbeiten:**

- Universitätsklinik (>800 Betten & Lehre/Forschung) ☐
- Maximalversorgung (>800 Betten) ☐
- Schwerpunktversorgung (501-800 Betten) ☐
- Basisversorgung (<= 500 Betten) ☐

**A8. Region der Einrichtung:**

- Großstadt (>= 100.000 Einwohner) ☐
- Stadt (5.000-99.999 Einwohner) ☐
- Landgemeinde (<5.000 Einwohner) ☐

# Teil B: Einschätzung zur Geschlechtersensibilität

Bitte beantworten Sie alle nachfolgenden Fragen.

**B1. Im Folgenden sind verschiedene Aussagen zum Thema "Geschlechtsunterschiede in der Pflege" aufgeführt.**

**Schätzen Sie bitte ein, wie gut die jeweilige Aussage Ihrer Meinung nach zutrifft und kreuzen Sie das entsprechende Kästchen an.**

| | Ich stimme ganz und gar nicht zu (--) | Ich stimme überwiegend nicht zu | Ich stimme teils zu/teils nicht | Ich stimme überwiegend zu | Ich stimme voll und ganz zu (++) |
|---|---|---|---|---|---|
| Ein fundiertes Wissen von Pflegekräften über Geschlechtsunterschiede verbessert die Qualität der pflegerischen Versorgung. | ☐ | ☐ | ☐ | ☐ | ☐ |
| Pflegekräfte sollten sich nur mit biologischen Unterschieden zwischen Männern* und Frauen* befassen. | ☐ | ☐ | ☐ | ☐ | ☐ |
| Bei nicht-geschlechtsspezifischen Beschwerden spielt das Geschlecht der Patient*innen keine Rolle. | ☐ | ☐ | ☐ | ☐ | ☐ |
| Pflegekräfte sollten sich weitestgehend auf rein pflegerische Aspekte von Gesundheitsbeschwerden von Männern* und Frauen* beschränken. | ☐ | ☐ | ☐ | ☐ | ☐ |
| Pflegekräfte müssen nicht wissen, was im Leben von Männern* und Frauen* vor sich geht, um pflegerische Versorgung leisten zu können. | ☐ | ☐ | ☐ | ☐ | ☐ |
| Unterschiede zwischen weiblichen Pflegekräften und männlichen Pflegekräften sind zu gering, um relevant zu sein. | ☐ | ☐ | ☐ | ☐ | ☐ |
| Gerade weil Männer* und Frauen* unterschiedlich sind, sollten Pflegekräfte alle Patient*innen gleich behandeln. | ☐ | ☐ | ☐ | ☐ | ☐ |
| Pflegekräfte, die sich mit Geschlechterunterschieden befassen, befassen sich nicht mit den wichtigen Themen. | ☐ | ☐ | ☐ | ☐ | ☐ |

| | Ich stimme ganz und gar nicht zu (--) | Ich stimme überwiegend nicht zu | Ich stimme teils zu/teils nicht | Ich stimme überwiegend zu | Ich stimme voll und ganz zu (++) |
|---|---|---|---|---|---|
| In der Kommunikation mit Patient*innen spielt es für die Pflegekräfte keine Rolle, ob die Patient*innen männlich oder weiblich sind. | ☐ | ☐ | ☐ | ☐ | ☐ |
| In der Kommunikation mit Patient*innen spielt es keine Rolle, ob die Pflegekraft männlich oder weiblich ist. | ☐ | ☐ | ☐ | ☐ | ☐ |
| Unterschiede zwischen männlichen und weiblichen Patient*innen sind so gering, dass Pflegekräfte sie kaum berücksichtigen können. | ☐ | ☐ | ☐ | ☐ | ☐ |
| Pflegekräfte sollten für eine effektive Behandlung Geschlechtsunterschiede in Krankheitsursachen und -folgen ansprechen. | ☐ | ☐ | ☐ | ☐ | ☐ |
| Es ist nicht notwendig bei der Schilderung von Beschwerden Geschlechterunterschiede zu berücksichtigen. | ☐ | ☐ | ☐ | ☐ | ☐ |

## Teil C: Fragen zur geschlechtersensiblen Pflege

Bitte bewerten Sie die nachfolgenden Aussagen, inwiefern Aspekte der Pflege Expertenstandards umgesetzt werden.

**C1. Expertenstandard Entlassungsmanagement in der Pflege:**

**Bei stationär aufgenommenen Patient*innen erfassen wir immer...**

| | Ja | Nein | Manchmal | Weiß nicht |
|---|---|---|---|---|
| das Alter. | ☐ | ☐ | ☐ | ☐ |
| die ethnische Zugehörigkeit. | ☐ | ☐ | ☐ | ☐ |
| das Geschlecht. | ☐ | ☐ | ☐ | ☐ |
| chronische Krankheiten. | ☐ | ☐ | ☐ | ☐ |

**C2. Expertenstandard Schmerzmanagement in der Pflege:**

**Östrogene und die Genetik der Frau können eine Rolle bei der Schmerzwahrnehmung und -prävalenz spielen.**

Ja ☐

Nein ☐

## C3. Expertenstandard Sturzprophylaxe in der Pflege:

| | Trifft nicht zu | Trifft eher nicht zu | Trifft eher zu | Trifft voll zu | Weiß nicht |
|---|---|---|---|---|---|
| Ich leite besondere Maßnahmen zur Sturzprophylaxe ein, wenn ein Prostatakrebs-Patient Androgenrezeptor-Inhibitoren einnimmt. | ☐ | ☐ | ☐ | ☐ | ☐ |

## C4. Expertenstandard Förderung der Harnkontinenz in der Pflege:

| | Trifft nicht zu | Trifft eher nicht zu | Trifft eher zu | Trifft voll zu | Weiß nicht |
|---|---|---|---|---|---|
| In unserer Einrichtung berücksichtigen wir den Wunsch von Patient*innen mit Harninkontinenz (sofern möglich), von einer gleichgeschlechtlichen Pflegeperson versorgt zu werden. | ☐ | ☐ | ☐ | ☐ | ☐ |
| In unserer Einrichtung werden Anschauungsmaterialien vorgehalten, die zur Beratung von Harninkontinenz-Patient*innen genutzt werden. | ☐ | ☐ | ☐ | ☐ | ☐ |
| In unserer Einrichtung stehen uns verschiedene Inkontinenzhilfsmittel zur Verfügung, die es uns ermöglichen, eine situationsgerechte und geschlechtsspezifische Auswahl zu treffen. | ☐ | ☐ | ☐ | ☐ | ☐ |
| Unsere Einrichtung stellt sicher, dass auch auf allen Herrentoiletten ausreichend große Eimer zum Abwurf von Inkontinenzvorlagen und -hosen unmittelbar neben dem WC stehen. | ☐ | ☐ | ☐ | ☐ | ☐ |
| Wir achten bei der Personalplanung darauf, dass auf einer Schicht (sofern vorhanden) möglichst männliches und weibliches Gesundheits- und Krankenpflegepersonal zusammenarbeiten. | ☐ | ☐ | ☐ | ☐ | ☐ |

## C5. Expertenstandard Förderung der Harnkontinenz in der Pflege:

**In unserer Einrichtung werden für bettlägerige Patient*innen Toilettenhilfen verwendet,...**

| | Trifft nicht zu | Trifft eher nicht zu | Trifft eher zu | Trifft voll zu | Weiß nicht |
|---|---|---|---|---|---|
| ...die speziell auf die Anatomie der Frau ausgerichtet sind. | ☐ | ☐ | ☐ | ☐ | ☐ |
| ...die speziell auf die Anatomie des Mannes ausgerichtet sind. | ☐ | ☐ | ☐ | ☐ | ☐ |

LimeSurvey

**C6. Expertenstandard Ernährungsmanagement zur Sicherung und Förderung der oralen Ernährung in der Pflege:**

| | Trifft nicht zu | Trifft eher nicht zu | Trifft eher zu | Trifft voll zu | Weiß nicht |
|---|---|---|---|---|---|
| Unser Haus bietet die Möglichkeit, Einflussfaktoren (z.B. biografische, religiöse und kulturelle) bei der Ernährung und den Tischsitten der Patient*innen zu berücksichtigen. | ☐ | ☐ | ☐ | ☐ | ☐ |

**C7. Frauen präsentieren sich bei einem akuten Myokardinfarkt häufiger mit Symptomen, wie abnorme Müdigkeit, Luftnot und Übelkeit als Männer.**

Ja ☐

Nein ☐

**C8. Bitte beantworten Sie die abschließende Frage:**

| | Sehr gut | Gut | Wenig | Wird gar nicht umgesetzt | Kann ich nicht beurteilen |
|---|---|---|---|---|---|
| Inwiefern wird aus Ihrer Sicht die geschlechtersensible Versorgung (z.B. die Wahrung der Intimsphäre von Patientinnen und Patienten; Respektierung der religiösen Zugehörigkeit, z.B. Raum zum Beten oder Berücksichtigung vom Migrationshintergrund, z.B. Sprachbarrieren oder Ernährungsgewohnheiten) auf dieser Station umgesetzt? | ☐ | ☐ | ☐ | ☐ | ☐ |

**C9. Optional: Welche Rolle spielt aus Ihrer Sicht das Geschlecht des Patienten oder der Patientin bei der Pflege-Patienten-Kommunikation?**

**C10. Gibt es noch etwas, was Sie zur Thematik anmerken möchten?**

**Anmerkungen (optional):**

**<u>Herzlichen Dank für Ihre Teilnahme!</u>**

**Wenn Sie Interesse an einer Zusammenfassung der Ergebnisse des letzten Teils der Befragung zu Inhalten aus den Expertenstandards zur geschlechtersensiblen Pflege haben, schreiben Sie bitte eine formlose E-Mail an: j.mollenhauer@figus.koeln, Tel.: 0221-29257382.**

**Judith Mollenhauer M.Sc. (wissenschafltiche Mitarbeiterin des figus-Instituts)**
